# The Utility of Maternal Point of Care Ultrasound on Labor and Delivery Wards

**DOI:** 10.3390/jcdd9010029

**Published:** 2022-01-14

**Authors:** Mohammed Algodi, Diana S. Wolfe, Cynthia C. Taub

**Affiliations:** 1 Department of Medicine, Division of Cardiology, Montefiore Medical Center, Albert Einstein College of Medicine, Bronx, NY 10467, USA; malgodi@montefiore.org; 2 Department of Obstetrics and Gynecology and Women’s Health, Division of Maternal Fetal Medicine, Montefiore Medical Center, Albert Einstein College of Medicine, Bronx, NY 10467, USA; dwolfe@montefiore.org; 3 Section of Cardiovascular Medicine, Heart and Vascular Center, Dartmouth-Hitchcock Medical Center, Geisel School of Medicine at Dartmouth, Lebanon, NH 03756, USA

**Keywords:** POCUS, labor and delivery

## Abstract

Point-of-care ultrasonography (POCUS) refers to limited bedside ultrasound used to evaluate patients for conditions specific to the scope of their practice. Given the benefits of its application, interest in its use is increasing. We aimed to review the literature and assess the potential feasibility of using POCUS of the heart and lungs in the field of obstetrics. We aim to describe its relevance and value as an adjunctive tool for critically ill obstetric patients on labor and delivery wards.

## 1. Introduction

Ultrasound imaging is a non-invasive and widely available modality to guide timely diagnosis and enhance therapeutic strategies. The advancements in technology over the past two decades have improved the portability of equipment, enabling ultrasound imaging to be performed at the bedside and thereby allowing physicians from all specialties to make treatment decisions or perform ultrasound-guided procedures. It is different from the standard, conventional ultrasound in that it is usually a limited and focused exam performed at the bedside. In 2004, the American Institute of Ultrasound in Medicine (AIUM) hosted a conference on compact ultrasonography, which is the idea of an “ultrasound stethoscope” as a rapid moving tool [1,2,3]. The Australasian Journal of Ultrasound in Medicine (AJUM) stated that POCUS is a study that is performed by a medical practitioner who uses ultrasound equipment to enhance and extend their own clinical examination of the patient and should be considered an extension of the physical examination [4,5]. POCUS is now widely recognized to complement the physical examination and serve as a safe interventional guidance at the bedside. Moreover, an ultrasound has become a routine approach when caring for critically ill patients including those on labor and delivery wards [6,7,8]. It has a wide range of applications, including but not limited to obtaining images of the thorax (lung and pleural), abdomino-pelvis, vascular, and cardiac organs. Guidelines on the use of POCUS have been established by the Societies of Emergency and Critical Care Medicine [9,10]. On labor and delivery wards, an ill parturient warrants a critical care consult which may require a POCUS of the maternal heart and lungs.

**Figure 1:**
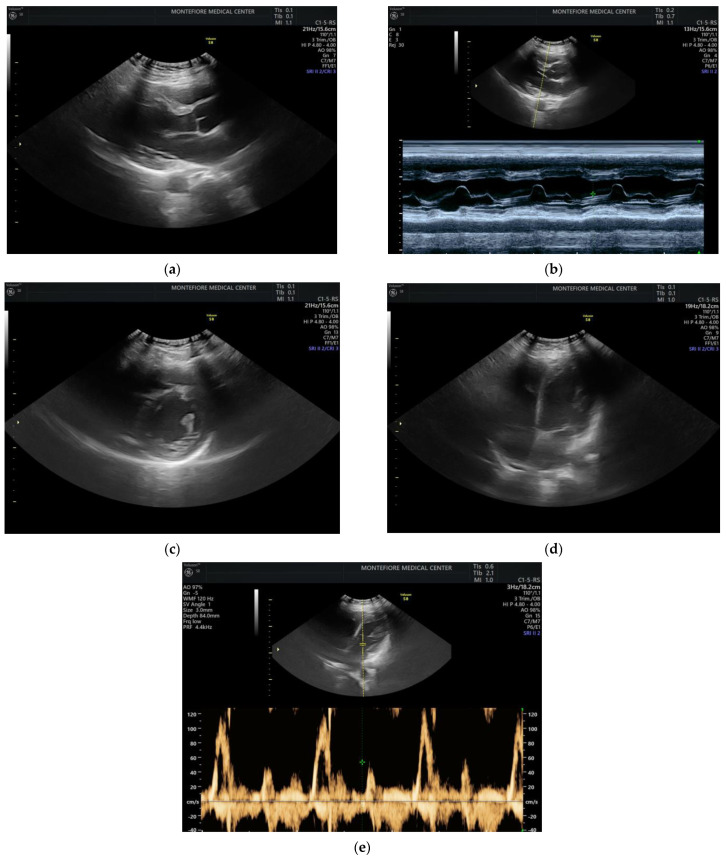
Performing cardiac POCUS on a pregnant patient using an OB probe may provide adequate diagnostic information. (**a**) Parasternal long axis view. (**b**) M-mode of the mitral valve. (**c**) Parasternal short axis view. (**d**) Apical 4-chamber view. (**e**) Pulse wave Doppler.

If obstetric providers were trained to perform a quick maternal cardiac and lung assessment for the most ill patients on the labor floor, it could facilitate the rapid management of these cases [11]. POCUS can be applied to obstetric and peri-partum patients to rule out pulmonary edema and other cardiac abnormalities. An ultrasound with an obstetric probe is readily available on the labor floor, making the POCUS exam quick and feasible (Figure 1) while awaiting additional imaging and critical care subspecialists.

## 2. Methods

In this review, a literature search was performed using Google Scholar and PubMed, capturing data from 2004 to 2020. Key words used for this literature search included POCUS, Labor and Delivery, and Echocardiography. Relevant studies were identified and discussed among authors, focusing on the relevance of POCUS in clinical settings and its potential utility on the labor and delivery unit. 

To assess the feasibility of performing cardiac POCUS using an available OB probe, we obtained verbal consent from a pregnant patient and acquired cardiac images using a curved OB ultrasound transducer (images as shown in Figure 1).

## 3. History and Evolution of POCUS

Patient care has been revolutionized by technologies such as POCUS and digitized information in nearly every medical specialty. Physicians had limited bedside tools for centuries, but with POCUS, we could visualize what we can only infer through palpation or auscultation. In the early twentieth century, the sinking of the Titanic followed by the start of World War I, led to the development of sonar, the application was broadened and developed to be first used in medicine in the 1940s Figure 2. During the 1950s, many pioneers advanced the field of medical ultrasound, marking the birth of obstetric and gynecologic ultrasound and establishing the field of echocardiography. Ultrasound technology continued to advance during the 1970s and 1980s with the development of more sophisticated transducers, along with refinements in image quality and began to be used in emergency care. This was a role that marked the beginning of the POCUS era, used mostly by surgeons and emergency medicine physicians, who started assessing trauma patients with ultrasound. POCUS has entered many specialty practices in the 1990s. The vital changes during the 2000s led to the portability and affordability of ultrasound machines which facilitated an exponential increase in use by all providers. Subsequently, several professional societies published guidelines on the use of POCUS including the American Institute of Ultrasound in Medicine (AIUM), American College of Emergency Physicians (ACEP), American Society of Echocardiography (ASE) and the Society of Critical Care Medicine (SCCM). Additionally, in the early 2000s, the Accreditation Council for Graduate Medical Education (ACGME) started to mandate that certain residency and fellowship programs in the United States include basic ultrasound education.

## 4. POCUS Application

An example of an application of POCUS for pregnant women has been illustrated during the COVID-19 pandemic. Exposure to staff and equipment was a concern while a rapid decision-making tool was needed to apply the World Health Organization (WHO) criteria and subsequent determination of level of care and respiratory treatment associated with their classification of the disease. A case series of eight infected pregnant women in Turkey who presented in respiratory distress had a lung POCUS [12]. It changed the management in seven of the eight patients with early detection of lung involvement in these COVID-19-infected pregnant women. When compared to other imaging studies such as computed tomography (CT) and chest X-ray (CXR), lung ultrasound (LUS) was substituted for chest CT in this vulnerable population of pregnant women. The authors report that during the pandemic, it has been suggested that obstetricians use LUS because they are already trained in ultrasound, reducing exposure to staff and accelerating time to diagnosis. Another case series from Italy reported four pregnant women with COVID-19 pneumonia detected with lung ultrasound prior to Polymerase chain reaction (PCR) results confirming their diagnosis. In [13], it was concluded that LUS is more efficient than CXR in this case series. LUS findings included irregular pleural lines, vertical artifacts, and large consolidations seen in one critically ill patient who was followed up with serial POCUS lung exams confirming improvement. While CT of the chest is the gold standard for COVID-19 pneumonia, serial exams impose a radiation risk to pregnant patients. Furthermore, the ASE statement on POCUS during the 2019 Novel Coronavirus Pandemic supports the use of POCUS to triage the dyspneic patient and determine what further imaging study is needed to best outline a patient’s plan of care [14,15,16,17].

## 5. Advantages of POCUS in Obstetrics Practice

### 5.1. Safety

According to the available evidence, exposure to diagnostic ultrasonography during pregnancy is safe [18]. In fact, sonogram is used throughout pregnancy for fetal monitoring. Maternal diagnostic ultrasounds are carried out throughout pregnancy when indicated. Therefore, the application of POCUS in maternal diagnostics is reasonable and is considered safe.

### 5.2. Clinical Application

POCUS is a vital tool in assessing many clinical conditions including, but not limited to, heart failure with decreased left ventricular ejection fraction (peri-partum cardiomyopathy), pleural effusion, pericardial effusion with or without cardiac tamponade, valvular heart disease, aortic aneurysm/aortic dissection, infective endocarditis, and pulmonary embolism with right heart strain. A study showed that POCUS is a valuable tool in the evaluation of preeclampsia with severe features such as pulmonary edema. Although the study was not designed to directly influence clinical management, the findings suggest that POCUS may serve as a useful adjunct to clinical examination for the managing these critically ill cases [19]. POCUS is an essential piece of equipment in the evaluation of alveolar interstitial syndrome, diagnosed based on comet tails. These are an ultrasound artifact that arises when ultrasound encounters a small air fluid interface, extending from the pleural line to the bottom of the screen, also known as “B lines”. In acute setting, B lines represent pulmonary edema, but they may also be encountered in acute respiratory distress syndrome. At the bedside, ultrasonography is highly sensitive, specific, and has been shown to be more accurate than auscultation or chest X-ray for identifying pleural effusion, consolidation, and alveolar interstitial syndrome, adding more diagnostic accuracy [20,21]. A reasonable number of case reports have been published emphasizing the use of POCUS to diagnose and assess various clinical conditions related to obstetric complications in pregnancy; for example, maternal abdominal hemorrhage in the case of uterine rupture.

### 5.3. Length of Stay and Cost Effectiveness

POCUS facilitates rapid triage of patients. In an analysis of length of stay in the emergency department for patients who received POCUS confirming live intrauterine pregnancy in the first trimester with pain and/or bleeding, the decrease in length of stay was most apparent for patients presenting during evening and nighttime hours [22]. A prospective study studied length of stay in the emergency department, comparing bedside ultrasonography to the conventional radiology department ultrasonography, concluding that POCUS resulted in a significant decrease in time to ultrasound and emergency department length of stay [23]. The mentioned advantages thereby carrying the potential to increase patient satisfaction. The literature suggests that POCUS has other advantages, such as improving quality and cost-effectiveness. This is a promising tool, but it requires dedicated training and experience to ensure safety and precision in making diagnoses.

## 6. Challenges and Opportunities

Although there are many advantages to POCUS, there are limitations. We outline here these challenges and strategies to overcome them.

### 6.1. Image Acquisition and Interpretation: ECHO Lab

POCUS of the maternal heart and lungs will potentially vary depending on the operator and ultrasound equipment. Therefore, when there is high suspicion of an abnormality, a formal echocardiogram or other radiographic study will be warranted. The POCUS exam is not intended to substitute a diagnostic test; its utilization is to expand the physical exam when formal testing cannot be performed instantly and while awaiting a critical care consult. Table 1 illustrates the characteristics and differences between the cardiac and obstetrics probes, shwoing their respective potential in clinical applications.

### 6.2. Litigation

Another limitation is the concern of litigious action and lawsuit cases against physicians acquiring bedside ultrasonography imaging. For example, the extent of lawsuits filed against emergency physicians over point-of-care emergency ultrasound was reported in the American Journal of Emergency Medicine in 2012; a total of one case in 20 years was filed against an emergency physician. It was over failure to perform ultrasound at the bedside, resulting in delayed or missed diagnoses [24]. This argued in favor of more wide-spread use of POCUS.

### 6.3. Documentation and Reimbursement

Among the barriers to implementing POCUS is documentation and billing. To address this concern, the Society of Hospital Medicine released a Position Statement in 2018 outlining a standard documentation. This should include the indication and type of ultrasound examination performed, date and time of the examination, patient identifying information, name of provider acquiring and interpreting the images, specific scanning protocols used, patient position, probe used, and findings. Whenever possible, documentation should be timely to facilitate communication with other providers. As mentioned in the statement, billing is supported through the AMA Current Procedural Terminology codes for “focused” or “limited” ultrasound examinations. The following points must be addressed for billing: (A) Images must be permanently stored. Specific requirements vary by insurance policy, though current practice suggests a minimum of one image demonstrating relevant anatomy and pathology for the ultrasound examination coded. (B) Proper documentation must be entered in the medical record. (C) Local institutional privileges for POCUS must be considered [25]. This is a snapshot of possible solutions and beyond the scope of this paper. On the labor and delivery unit, we do not intend to bill for POCUS, rather its application to decrease time to diagnosis and treatment is our main goal. In an environment of bundled payment and value-based healthcare, the use of POCUS steers away from the fee-for-service model.

### 6.4. Training and Quality Assurance

Several specialty organizations including emergency medicine, critical care, anesthesiology, obstetrics, and cardiology published guidelines and statements regarding quality assurance (QA) in POCUS. The aim is to ensure that physicians maintain basic competency to make bedside decisions. Oversight includes ensuring that providers using POCUS are appropriately trained, using the equipment correctly, and documenting properly. Some programs have implemented mechanisms to review and provide feedback on image acquisition, interpretation, and clinical integration [26,27,28]. Evolving training and QA solutions driven by artificial intelligence, machine learning, and deep learning may empower POCUS users to gain confidence rapidly.

## 7. Conclusions

Ultrasound has many advantages over other imaging modalities, making it well-suited to the acute care setting. The ultrasound equipment is portable and relatively affordable, the images may be obtained and interpreted in real time and the procedure is proven to be safe. This is particularly relevant in the field of obstetrics, with the highest potential for reduction in morbidity and mortality on Labor and Delivery wards. POCUS may provide incremental value to improve our diagnostic and therapeutic capabilities. It has the potential to improve patient safety, decrease length of stay in the hospital and reduce healthcare costs. As POCUS spreads throughout the profession of medicine, it is imperative that physicians engage in research to direct its appropriate use. Active collaboration between expert users, educators, and allied healthcare providers to create future generations of clinicians that will be equipped to use POCUS is ongoing. We acknowledge that there are limitations and have made suggestions to overcome these barriers. This encompasses the incorporation of appropriate training and perform routine operator evaluations to ensure that the adequate and appropriate skill level is maintained.

## Figures and Tables

**Figure 2:**
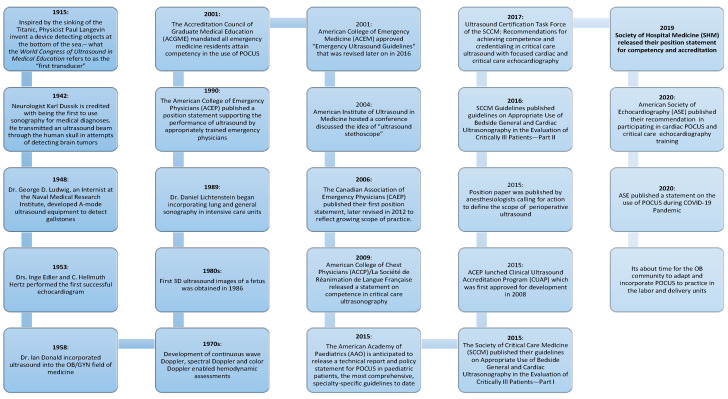
Historical timeline of POCUS evolution. The timeline highlights some important events over the past century. Emergence of POCUS guidelines from major medical societies and wide-spread use in clinical practice shaped the way we practice medicine today.

**Table 1:** Comparison of cardiac and obstetrics transducers: significant overlaps of transducer frequencies and recommended exam types are listed.

Transducers		
	Cardiac	Obstetrics
Type	Phased array	Curved
Footprint	24 × 17 mm	61 × 17 mm
	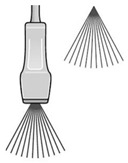	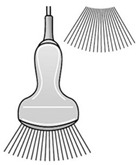
Frequency	1.1–4.8 MHz	1.4–5.0 MHz
Imaging depth	35 cm	30 cm
Exam Types	Abd, FAST, LUNG, Renal, Adult Echo, Ped Echo, TCD	Abd, Renal, Bowel, FAST, Lung, OB, Early OB, OB (Adv), Fetal Echo, GYN, Venous, Arterial, Pelvis, Spine
Images	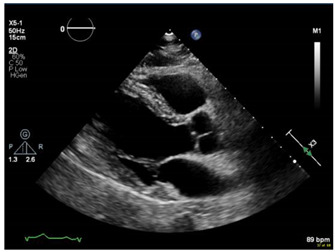	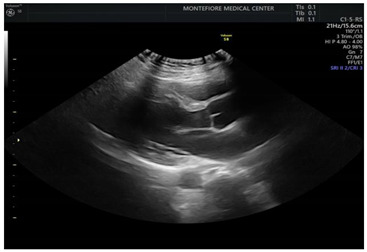

## Data Availability

Not applicable.

## References

[1] Moore C.L., Copel J.A. (2011). Point-of-Care Ultrasonography. N. Engl. J. Med..

[2] Greenbaum L.D., Benson C.B., Nelson L.H.I.I.I., Bahner D.P., Spitz J.L., Platt L.D. (2004). Proceedings of the Compact Ultrasound Conference sponsored by the American Institute of Ultrasound in Medicine. J. Ultrasound Med..

[3] Alpert J.S., Mladenovic J., Hellmann D.B. (2009). Should a hand-carried ultrasound machine become standard equipment for every internist?. Am. J. Med..

[4] AIUM Ultrasound Practice Forum, 2010: Point-of-Care Use of Ultrasound. http://www.aium.org/advertising/2010Forum.pdf.

[5] Australasian Society for Ultrasound in Medicine (ASUM) (2017). Discussion Paper: Definition of Point of Care Ultrasound (POCUS). https://www.asum.com.au/standards-of-practice/.

[6] Collins K., Collins C., Kothari A. (2019). Point-of-care ultrasound in obstetrics. Australas. J. Ultrasound Med..

[7] Blanco P. (2015). A traditional paradigm vs. an ultrasound-supported paradigm in emergency and critical care medicine: A crisis of the mind is needed. J. Emerg. Med..

[8] Álvarez-Fernández J.A., Núñez-Reiz A. (2015). En representación del Club de Ecografía UCI Madrid de la SOMIAMA Clinical ultrasound in the ICU: Changing a medical paradigm. Med. Intensiva..

[9] Frankel H.L., Kirkpatrick A.W., Elbarbary M., Blaivas M., Desai H., Evans D., Summerfield D.T., Slonim A., Breitkreutz R., Price S. (2015). Guidelines for the Appropriate Use of Bedside General and Cardiac Ultrasonography in the Evaluation of Critically Ill Patients-Part I: General Ultrasonography. Crit Care Med..

[10] Levitov A., Frankel H.L., Blaivas M., Kirkpatrick A.W., Su E., Evans D., Summerfield D.T., Slonim A., Breitkreutz R., Price S. (2016). Guidelines for the Appropriate Use of Bedside General and Cardiac Ultrasonography in the Evaluation of Critically Ill Patients-Part II: Cardiac Ultrasonography. Crit Care Med..

[11] Sapoval J., Carter R.E. (2019). Ultrasound Biophysical Profile. StatPearls.

[12] Yassa M., Mutlu M.A., Kalafat E., Birol P., Yirmibeş C., Tekin A.B., Sandal K., Ayanoğlu E., Yassa M., Kılınç C. (2020). How to perform and interpret the lung ultrasound by the obstetricians in pregnant women during the SARS-CoV-2 pandemic. Turk. J. Obstet. Gynecol..

[13] Buonsenso D., Raffaelli F., Tamburrini E., Biasucci D.G., Salvi S., Smargiassi A., Inchingolo R., Scambia G., Lanzone A., Testa A.C. (2020). Clinical role of lung ultrasound for diagnosis and monitoring of COVID-19 pneumonia in pregnant women. Ultrasound Obs. Gynecol..

[14] Khoury R., Bernstein P.S., Debolt C., Stone J., Sutton D.M., Simpson L.L., Dolan S.M. (2020). Characteristics and Outcomes of 241 Births to Women with Severe Acute Respiratory Syndrome Coronavirus 2 (SARS-CoV-2) Infection at Five New York City Medical Centers. Obstet. Gynecol..

[15] Johri A.M., Galen B., Kirkpatrick J.N., Lanspa M., Mulvagh S., Thamman R. (2020). ASE Statement on Point-of-Care Ultrasound during the 2019 Novel Coronavirus Pandemic. J. Am. Soc. Echocardiogr..

[16] American College of Emergency Physicians (2009). Emergency ultrasound guidelines. Ann. Emerg. Med..

[17] Bennett C.E., Samavedam S., Jayaprakash N., Kogan A., Gajic O., Sekiguchi H. (2018). When to incorporate point-of-care ultrasound (POCUS) into the initial assessment of acutely ill patients: A pilot crossover study to compare 2 POCUS-assisted simulation protocols. Cardiovasc. Ultrasound.

[18] Torloni M.R., Vedmedovska N., Merialdi M., Betrán A.P., Allen T., González R., Platt L.D. (2009). Safety of ultrasonography in pregnancy: WHO systematic review of the literature and meta-analysis. Ultrasound Obs. Gynecol..

[19] Ortner C.M., Krishnamoorthy V., Neethling E., Flint M., Swanevelder J.L., Lombard C., Fawcus S., Dyer R.A. (2019). Point-of-Care Ultrasound Abnormalities in Late-Onset Severe Preeclampsia: Prevalence and Association with Serum Albumin and Brain Natriuretic Peptide. Anesth. Analg..

[20] Liteplo A.S., Marill K., Villen T., Miller R.M., Murray A.F., Croft P.E., Capp R., Noble V.E. (2009). Emergency Thoracic Ultrasound in the Differentiation of the Etiology of Shortness of Breath (ETUDES): Sonographic B-lines and N-terminal pro-brain-type natriuretic peptide in diagnosing congestive heart failure. Acad. Emerg. Med..

[21] Lichtenstein D., Goldstein I., Mourgeon E., Cluzel P., Grenier P., Rouby J.J. (2004). Comparative diagnostic performances of auscultation, chest radiography, and lung ultrasonography in acute respiratory distress syndrome. Anesthesiology.

[22] Blaivas M., Sierzenski P., Plecque D., Lambert M. (2000). Do Emergency Physicians Save Time When Locating a Live Intrauterine Pregnancy with Bedside Ultrasonography?. Acad. Emerg. Med..

[23] Wilson S.P., Connolly K., Lahham S., Subeh M., Fischetti C., Chiem A., Aspen A., Anderson C., Fox J.C. (2016). Point-of-care ultrasound versus radiology department pelvic ultrasound on emergency department length of stay. World J. Emerg. Med..

[24] Blaivas M., Pawl R. (2012). Analysis of lawsuits filed against emergency physicians for point-of-care emergency ultrasound examination performance and interpretation over a 20-year period. Am. J. Emerg. Med..

[25] Soni N.J., Schnobrich D., Benji K.M., Tierney D.M., Trevor P.J., Dancel R., Joel C., Renee K.D., Gregory M., Anjali B. (2019). Point-of-Care Ultrasound for Hospitalists: A Position Statement of the Society of Hospital Medicine. J. Hosp. Med..

[26] (2016). American College of Emergency Physicians Policy Statement: Emergency Ultrasound Guidelines. https://www.acep.org/Clinical---Practice-Management/ACEP-Ultrasound-Guidelines/.

[27] Mayo P.H., Beaulieu Y., Doelken P., Feller-Kopman D., Harrod C., Kaplan A., Oropello J., Vieillard-Baron A., Axler O., Lichtenstein D. (2009). American College of Chest Physicians/La Societe de Reanimation de Langue Francaise statement on competence in critical care ultrasonography. Chest.

[28] (2018). AIUM–ACR–ACOG–SMFM–SRU Practice Parameter for the Performance of Standard Diagnostic Obstetric Ultrasound Examinations. J. Ultrasound Med..

